# The composite dataset of the present-day Infralittoral Prograding Wedges (IPWs) in the inner continental shelf of the Campania region (Central-Eastern Tyrrhenian Sea)

**DOI:** 10.1016/j.dib.2022.108484

**Published:** 2022-07-24

**Authors:** Francesca Budillon, Ines Alberico, Sabrina Amodio, Pasquale Contestabile, Sara Innangi

**Affiliations:** a Istituto di Scienze Marine, ISMAR, Consiglio Nazionale delle Ricerche, Calata Porta di Massa, 80133 Napoli Italy; b DiST - Dipartimento Scienze e Tecnologie, Università degli Studi di Napoli Parthenope, 80143 Napoli Italy; c Dipartimento Ingegneria, Università degli Studi della Campania Luigi Vanvitelli, 81031 Aversa Italy

**Keywords:** Marine and terrestrial DEM, Submarine depositional terraces, Morphometric indices, Significant wave high, Effective fetch, Southern Italy, CRID, Campania Region IPW Dataset, DEM, Digital Elevation Model, EF, Effective fetch, FSA, Foreset slope angle, GF, Geographical fetch, H_s_, Significant wave height, H_S,50_, Significant wave height at the 50 m isobath, H_S,ROP_, Significant wave height at the ROP, IPW, Infralittoral Prograding Wedge, RSL, Relative Sea Level, ROP, Rollover Point, T_p_, Peak wave period, T_R_, Time recurrence, TSA, Topset slope angle

## Abstract

This article reports on the dataset gathered following the census of 83 present-day Infralittoral Prograding Wedges (IPWs), surveyed on the inner continental shelf of the Central-Eastern Tyrrhenian Sea. The purpose of the census was to explore their bathymetric range and assess the observational laws governing this variability. The ensued dataset (Campania Region IPW Dataset, CRID) includes geographic, topographic and morpho-bathymetric indices, descriptive of each IPW and more, the exposure of each IPW to wave forcing (Geographical fetch, Effective fetch and extreme significant wave height, H*_S_*). In this work, histograms contribute to describe all the variables and highlight the dominant features of each IPW. Location maps univocally links the geographic position of each IPW to the appropriate attribute record in the dataset. Further, thematic maps illustrate eight wave fields obtained by offshore-to-nearshore transformation by as many sea states scenarios with 200-year return period. Such wave fields are used as sources for significant wave height representing wave conditions over each IPW.

This dataset could be implemented with new measures at a broader scale, by following analogue procedures for measurements, to enlarge the observational scale on IPWs and improve the numerical models which might eventually derive by the analysis of this dataset.

## Specifications Table

**Table:** 

Subject	Earth and Planetary sciences; Engineering; Data Science
Specific subject area	Coastal physiography; morpho-bathymetry; coastal exposure to wave forcing; extreme wave climate; data mining; statistical analysis
Type of data	**Worksheet,** **Graph,** **Figure,** **Maps**
How the data were acquired	The Campania Region IPW Dataset (CRID) has been acquired from several data sources. The morphological indices of the 83 IPWs have been drawn from marine and terrestrial DEMs by applying geospatial algorithms available in GIS- environment (Global Mapper ®, ArcMap, 10.8). The "coastline elevation in the backshore" 100 m inland (m, asl) was measured from terrestrial DEM [1]. "Effective Fetch (EF)" and "Geographical Fetch (GF)", here considered as proxies of IPWs exposure to wave forcing, have been calculated by using GEBCO_2020 (Grid General Bathymetric Chart of the Oceans, 2020) [2] and the geographical location of IPWs. EF namely, is the portion of sea which generates wind-waves and is computed by considering both length and width of the generation area. It was calculated in a GIS environment (ArcMap, 10.8) by applying a procedure based mainly on viewshed and drawing line algorithms [3]. GF is a simplified parameter of the wave exposure and was measured along a single direction at right angles to the IPW edge [4]. Raw offshore wave data were supplied by pitch-roll type directional buoys operating off the Island of Ponza (central Tyrrhenian Sea). Available records of buoys of Ponza are from 1 July 1989 to 31 December 2014 as a part of the Italian Wave Network [5], [6], [7]. An extreme value analysis of offshore wave data was carried out, by selecting a value of the return period, *T_R_,* of 200 years and deriving extreme sea states in front of each IPW. Values of "*H_S_*" were extracted at the 50 m-isobath (*H_S,50_*) and at each IPW edge (*H_S,ROP_*). Finally, the "relative sea level variation" at each IPW site in the last 2 ky was collected by literature references.
Data format	Raw, Analyzed, Filtered
Description of data collection	The CRID records measures derived by a detailed geomorphological analysis of shallow water and terrestrial DEM along the Campania Region (southern Italy). and waves analysis of temporal series of offshore wave data from the Ponza Buoy (central Tyrrhenian Sea). [7]
Data source location	• Istituto di Scienze Marine, ISMAR-CNR Naples, Italy. • Dipartimento Ingegneria, Università degli Studi della Campania Luigi Vanvitelli, Aversa (Caserta Province), Italy. • The collected dataset is included in an area with Latitude and Longitude of vertex at 41°08.3360’N, 013°43.1143’E; 39°54.0000’N, 015°45.1621’E.
Data accessibility	• [1] Terrestrial DEM is available at https://tinitaly.pi.ingv.it/Download_Area2.html • [2] Bathymetric data used for calculating EF are available at GEBCO_2020 Grid General Bathymetric Chart of the Oceans, https://www.gebco.net/data_and_products/gridded_bathymetry_data/. • [7] Offshore wave data from Ponza's wave buoy are available online at: http://dati.isprambiente.it/sparql. The data can be queried through the SPARQL endpoint made available according to all the principles defined by the RDF standard. In the field “Query Text”, insert the following query chaining for the dataset from 2002 to 2009: PREFIX: <http://dati.isprambiente.it/ontology/core#>PREFIX gn: <http://www.geonames.org/ontology#>PREFIX rdfs: <http://www.w3.org/2000/01/rdf-schema#>PREFIX dcat: <http://www.w3.org/ns/dcat#>PREFIX purl: <http://purl.org/dc/terms/>select distinct ?station ?period ?csvUrl where {#### Definire Parametro, Luogo, Dataset e Periodo?parameter a:Wave.?place rdfs:label "Ponza".?dataset rdfs:label "Dataset RON"@it.FILTER (str(?period) >= '2002-01′ AND str(?period) <= '2009-11′).?parameter gn:nearbyFeature ?place.?collection a:MeasurementCollection;:measurementPeriod ?period;:isDataOf ?parameter;:generatedBy ?instrument;purl:isPartOf ?dataset;dcat:downloadURL ?csvUrl.?instrument:placedOn ?stat.?stat rdfs:label ?station.} ORDER BY ?period. Instead, for the dataset from 2014 to 2019, insert the following query text: PREFIX: <http://dati.isprambiente.it/ontology/core#>PREFIX gn: <http://www.geonames.org/ontology#>PREFIX rdfs: <http://www.w3.org/2000/01/rdf-schema#>PREFIX dcat: <http://www.w3.org/ns/dcat#>PREFIX purl: <http://purl.org/dc/terms/>select distinct ?station ?period ?csvUrl where {#### Definire Parametro, Luogo, Rete?parameter a:Wave.?place rdfs:label "Ponza".?network rdfs:label "Rete Ondametrica Nazionale (2009-2014)".?parameter gn:nearbyFeature ?place.?collection a:MeasurementCollection;:measurementPeriod ?period;:isDataOf ?parameter;:generatedBy ?instrument;dcat:downloadURL ?csvUrl.?instrument:placedOn ?stat.?stat rdfs:label ?station.?stat purl:isPartOf ?network.} ORDER BY ?period • [8] Bathymetric data are available at EMODnet Bathymetry Consortium (2020): EMODnet Digital Bathymetry (DTM), https://doi.org/10.12770/bb6a87dd-e579-4036-abe1-e649cea9881a. • The IPWs dataset (CRID) is hosted in Supplementary Material as *.xls* file.
Related research article	F. Budillon, S. Amodio, I. Alberico, P. Contestabile, M. Vacchi, S. Innangi, F. Molisso, Present-day infralittoral prograding wedges (IPWs) in the Central-Eastern Tyrrhenian Sea: critical issues and challenges to their use as geomorphological indicators of sea level. Marine Geology, 450, 106821. https://doi.org/10.1016/j.margeo.2022.106821

## Value of the Data

• The data here presented allowed us to derive the observational law which governs the depth of the present-day infralittoral prograding wedgesin central-eastern Tyrrhenian Sea.
• The study aims to provide clues to solve a non-trivial issue that deals with the reliability of IPWs as proxies for sea level.
• Specifically, the reader could test the same procedure in different localities to widen the survey at a broader scale and strenghten the reliability of the observational law.

## 1 Data Description

**Fig. 1:**
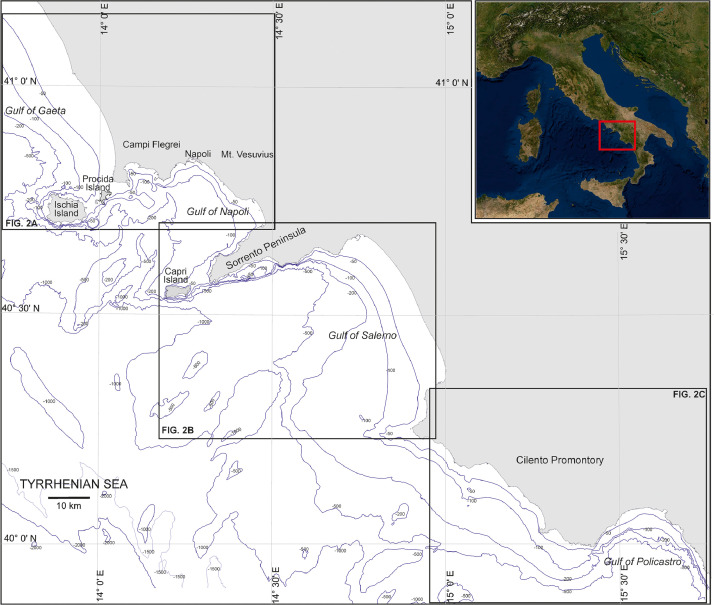
The dataset described in this article is relative to a sector of Southern Italy, facing the Central-Eastern Tyrrhenian Sea (Campania region). The red inset shows the location of the study area at a national scale.

**Fig. 2:**
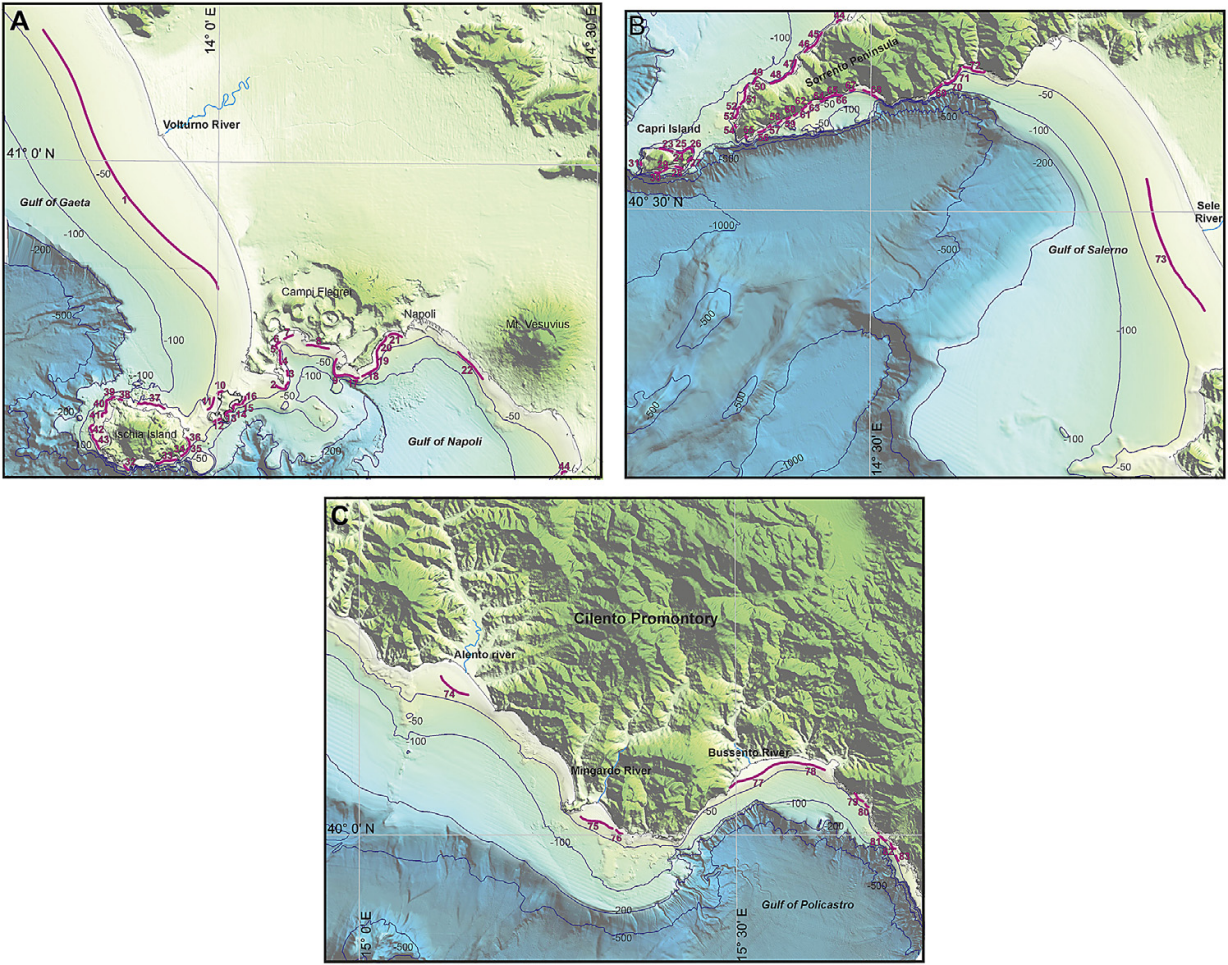
Location of the surveyed IPWs edges (purple lines) and related ID off A) Gulf of Gaeta, Pozzuoli Bay, Gulf of Napoli, Ischia and Procida Islands; B) Sorrento Peninsula, Capri Island and Gulf of Salerno; C) Cilento Promontory and Gulf of Policastro.

A census of 83 near-shore Infralittoral Prograding Wedges (IPWs) was realized on the inner continental shelf of the Campania Region (Table 1 in Supplementary Material), encompassing a coastline about 480 km long (Fig. 1). The location of each IPW, sequentially numbered, so as being univocally linked to its relative indices, is shown in figure 2.

Namely, the Campania Region IPW Dataset (CRID, Table 1 in Supplementary Material) includes for each IPW numerical indices, descriptive of morphometry, geography and wave climate, and a classification of the coast typology.

**Fig. 3:**
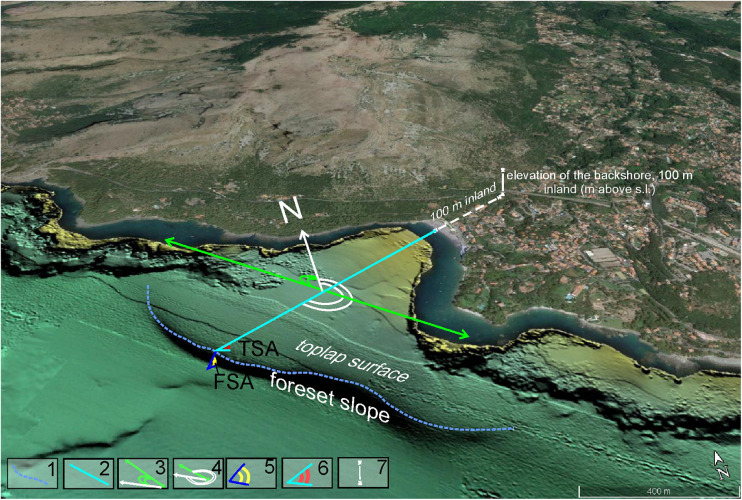
Measurements of morphometric parameters of IPWs: 1) IPW edge (m) along which the averaged ROP depth and length are calculated; 2) Distance of the terrace edge from the coastline; 3) Orientation (°N); 4) Direction (°N); 5) Foreset slope angle (FSA); 6) Toplap slope angle (TSA); 7) Elevation of the coast (m above sea level, asl) measured 100 m inland of the coastline.
• coastal physiography – described by four main coastal morpho-types: rectilinear rocky cliffs, rocky promontory, inlet, open coast shoreline [2]; the occurrence of pocket beach in the inlets is also reported;
• IPW ROP depth – the depth of the break-in-slope of the last-formed rollover point (ROP depth, meters below sea level); range: -10.60 m / -38 m;
• IPW edge-coast distance (m) – the length between the coastline and the terrace edge (Fig. 3); range: 0.07 - 6.6 km;
• IPW length (km) – alongshore extension of the terrace edge (Fig. 3); range: 0.2 - 31 km;
• Foreset Slope Angle (FSA, ° and %) – the foreset steepness measured in the direction of terrace progradation (Fig. 3); range: 0.7°- 20.8°;
• Toplap Slope Angle (TSA, ° and %) – the topset steepness measured in the direction of terrace growth (Fig. 3); range: 0.13°- 12.20°;
• direction – the main trend (°N) of terrace growth (Fig. 3); range: 0° - 340°;
• orientation – the main trend (°N) of terrace length (Fig. 3); range: 26° - 350°;
• Geographical Fetch (GF) (km) – the maximum length of the portion of the sea from which waves possibly come; this measurement was taken perpendicularly to the main orientation of the IPW; range: 3-643 km;
• Effective Fetch (EF) (km) – the exposition of each IPW coastal segment to the wave forcing, in other words the portion of sea which generates wind-waves; range: 4.40 – 617 km;
• H*_S,50_* – the significant wave height of a storm sea state with return periods T*_R_* = 200 years extracted at the 50 m-isobath in front of each IPW; range: 1.50 – 10.8 m;
• H*_S,ROP_* – the significant wave height of a storm sea state with return periods T*_R_* = 200 years extracted at the ROP depth of each IPW; range: 0.8-10.50 m;
• Late Holocene vertical movements trends – the vertical ground movement in terms of uplifting, subsiding or stable trends since from the Late Holocene;
• Relative Sea Level (RSL) at about 2 ky BP (m, bsl) – the local palaeo-sea level at 2 ky BP nearby each IPW and based on sea-level indicators of archeological, geomorphological, sedimentological and biological origin with respect to the present sea level; range: -1 m / -4.5 m;
• Bibliographic references for RSL values over the last 2 ky (see reference list in Supplementary Material).

• IPW_ID (progressive number );
• geographical coordinates (latitude and longitude);
• coastal orientation – averaged exposure of the backshore to a main geographical direction (N, NE, SE, S, SW, NW);
• elevation of the coastline in the backshore (m above sea level), (Fig. 3);

In Fig. 3 is graphically shown how the morphological indices on each IPW were measured.

**Fig. 4:**
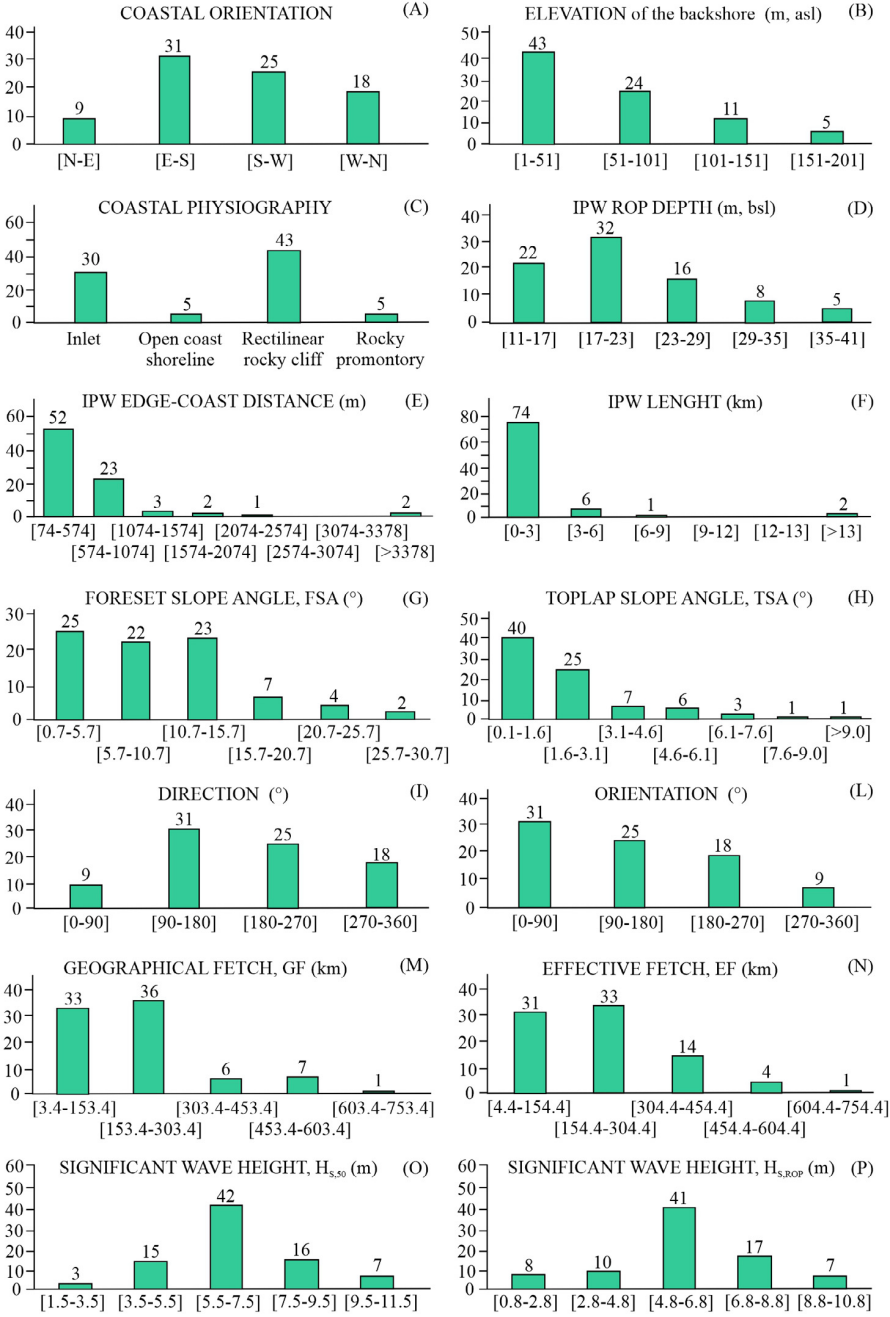
Frequency distribution of indices: the majority of IPWs indices features one predominant frequency class, whereas Coastal orientation, GF and EF show two predominant frequency classes; only FSA has three main frequency classes.

All the indices are then represented by frequency histograms (Fig. 4) to depict the distribution of their values. These diagrams show that: A) 56 IPWs develop off coasts mostly directed towards the SE and SW sectors; B) 43 IPWs lie off backshores that do not exceed 50 m asl; C) 43 IPWs develop offshore of rectilinear rocky cliffs (43), whereas 30 develop off inlets; D) 54 IPWs have ROP depths between 11 and 23 m; E) 52 IPWs are less than 574 m distant from the coast ; F) 74 IPWs are not longer than 3 km; G) FSA values between 0.7° and 15.7° are the most common and feature 70 IPWs; H) 65 IPWs show TSA ranging between 0.1° and 3.1°; I) 56 IPWs are directed between 90° and 270°N; L) 56 IPWs have an orientation between 0° and 180°; M) 69 IPWs and N) 64 IPWs show GF and EF within 300 km, respectively; O) 42 IPWs are exposed to H_S,50_ between 5.5 and 7.5 meters, while P) 41 IPWs are exposed to the H*_S,ROP_* ranging 4.8 and 6.8 meters.

## 2 Experimental Design, Materials and Methods

### 2.1 Morphological and geographical indices

Morphological and geographical indices (Table 1 in Supplementary Material) have been acquired from digital elevation models (DEM) of terrestrial coastal zone [1] and marine domanis by applying geospatial algorithms available in GIS-environment tools. The original regional-scale marine DEM merges different data sets with spatial resolution of grid cell varying from 5 m -side to of around 30 m –side [3]. In very shallow waters, the DEM is composed merging single and multibeam beam echo soundings (SBES and MBES) developed by IAMC CNR (now ISMAR CNR). Interpolations have been performed for the single beam bathymetric dataset mainly in the range -2/-12 m, where swath bathymetric data were not always available. Low-resolution bathymetric data are available at EMODnet Digital Bathymetry  [8].

The IPW indices measured by DEM (Fig. 3) are:

- Elevation in the backshore represents a numerical descriptor of the coastal morpho-type; this measurement is extracted from terrestrial DEM [1], by picking up the altitude at 100 m distance inland from the coastline, in the opposite direction of the terrace growth;
- IPW ROP depth is the average value of the water column, taken along-strike the IPW edge and computed by geospatial algorithms available in GIS-environment (Global Mapper ®, tool analysis measurements), and it is expressed in m bsl;
- IPW edge-coast distance is measured by geospatial algorithms available in GIS-environment (Global Mapper ®) along the orthogonal line depicted from coastline to the edge of the terrace and it is expressed in m;
- IPW length is the measure of the linear extent of the IPW edge (expressed in km);
- FSA is the foreset slope angle calculated downslope from the terrace edge median point along the maximum deep of the slope and is computed by geospatial algorithms available in GIS-environment (Global Mapper ®, path profile, sub-path info). This value is expressed in ° and percentage %;
- TSA is the toplap slope angle along the maximum deep in the direction of terrace growth and is calculated by geospatial algorithms available in GIS-environment (Global Mapper ®, path profile, sub-path info). This value is expressed in ° and percentage %;
- IPW direction is the angle between the segment along the growth direction of the terrace and the North (it is complementary to Orientation) and it is expressed in °;
- IPW orientation is the angle of the segment that joins the two extreme points of the terrace edge respect to the North, and it is expressed in °;
- GF is automatically obtained by an algorithm that measures the length, along a single direction at right angle to the IPW orientation, from the terrace edge to the nearest opposing coastline; it is expressed in km;
- EF is calculated in a GIS environment (ArcMap, rel 10.8). The input data are the GEBCO_2020 Grid, a continuous global terrain model of oceans and land with a spatial resolution of 15 arc seconds [2] and the geographical location of IPWs. The Effective Fetch, measured in km, represents a more refined version of GF, initially considered in [4]. EF is calculated by applying the following formula, derived by equation:
  $$\text{EF}=\frac{\sum_{i}GF_{i}\times \text{co}s^{n}\theta_{i}}{\sum_{i}\text{co}s^{n}\theta_{i}}$$

where $\theta_{i}$ is set at 10°; $GF_{i}$ is the geographical fetch along the seven directions; n is a coefficient proportional to the load attributed to the $GF_{i}$ (in our case n=2) [9].

### 2.2 Wave climate indices

Raw offshore wave data were supplied by pitch-roll type directional buoys operating off the Island of Ponza (Central Tyrrhenian Sea). The records are available since 1st July 1989 [5], [6], [7], as a part of the Italian Wave Network. From 1989 to about 2002, the wave buoys collected 30 min of wave measurements every 3 h, but when in presence of wave heights greater than 1.5 m, the measurements were continuous. From 2002 to 31 December 2014, the wave measurements have always been continuous and the wave characteristics parameters refer to 30-min time intervals. The dataset comprises the wave height computed on the zero-order moment of spectral function. (H*_m0_*), the mean wave period (T*_m_*) and the mean wave direction (*Dir*). For non-breaking waves, it can be assumed that H*_m0_* ≈ H*_S_*.

A pre-processing phase focused on a gross stochastic error detection was applied. The data processing was firstly regarded the missing data problem. Missing values reduce the representativeness of the sample. Moreover, it can severely disturb the conclusions drawn from the data. About 10% of missed data of about 20 years of observation have been recognized. In order to get a conservative estimation in case of lack in the time series, missed data or values of wave height less than 0.2 m for several hours were considered as errors and removed. In addition, to test the sensitivity of the results, H*_S_* = 1 m and 2 m were also used. The sensitivity analysis showed that the estimated wave energy flux does not differ substantially if wave heights of 1 m or 2 m were used to fill the missed data. After the regularization procedure, taking into account missing data, unrealistic calm conditions and spikes, a virtual geographical transposition of the time series was applied, creating a virtual buoy located offshore of the Gulf of Napoli (40°29′45.06″ N; 13°47′46.70″ E; depth of 1037 m). For details in the application of the method, see [10].

To define the intensity of rare storm conditions (i.e. hours of Mediterranean hurricane, otherwise known as “medicane”), according to current coastal engineering practice, Extreme Value Analysis were carried out. The last is a branch of statistics dealing with the extreme deviations from the median of probability distributions. Knowledge of the value of an extreme event for a given return period T*_R_* is the main result of the Extreme Value Analysis. Therefore, extreme events are described in terms of function H*_S_*(T*_R_*) which links the significant wave height of a sea state with different return periods T*_R_*. To produce a set of offshore extreme significant wave height values, the Peak Over Threshold (POT) method was followed. According to current ocean engineering practice, the Weibull distribution was adopted as extreme value distribution:

$$F\left(H_{S}\right)=1-exp\left\{-{\left[\left(H_{S}-b\right)/a\right]}^{c}\right\}$$

where a, b and c are the scale, position and shape parameters, respectively. In this work, these parameters were estimated by means of the last squares method. Then, the *H_S_* value for a given return period (in years) is computed as:

$$H_{S}\left(T_{R}\right)=b+a{\left[\text{ln}\left(\tau T_{R}\right)\right]}^{1/c}$$

The τ T*_R_* term derives from the POT techniques, where τ extreme values are considered on average for each observation year.

The value of T*_R_* should be long enough to be representative of wave conditions over the IPWs lifetime but, at the same time, compatible with the length of the original time series used to perform the statistical analysis. These considerations led to the selection of a T*_R_*=200 years. The extreme value analysis has been repeated considering three directional wave sectors (i.e., 70°-190° N; 190°-250° N; 250°-320° N). Consequently, three H*_S_* (T*_R_*) with the following values: 6.5 m, 8.3 m and 10.8 m, respectively, were obtained. Since a sea state is defined by a triple of H*_S_*, peak wave period (T*_p_*) and wave direction (*Dir*), the following procedure was adopted. The peak wave period T*_p_*, related to each extreme value of H*_S_* was calculated as:

$$T_{p}=8.5\pi \sqrt{\frac{H_{s}}{4g}}$$

**Fig. 5:**
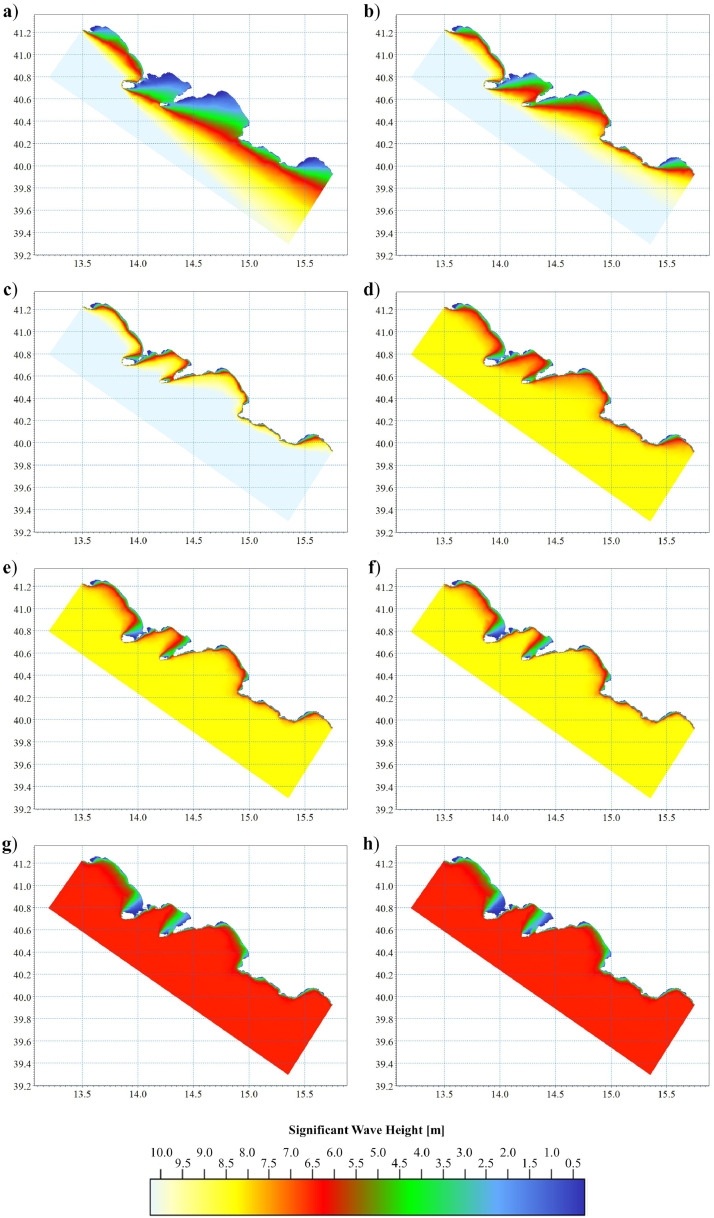
Wave field of significant wave height for extreme scenarios with 200-year return period coming from eight wave sectors. Letters refer to such scenarios, as denoted in paragraph 4.2 of [3].

For the wave direction, eight representative values of the whole wave sector were selected: 140, 175, 190, 215, 240, 250, 285 and 320° N. The eight extreme sea states are reported in [3]. These extreme sea states were used to force the wave propagation model. As results, eight wave fields were obtained. The results are graphically represented by pictures assembled in Fig. 5. From the eight datasets, it has been selected the most energetic wave field in respect to each IPW, i.e. the highest value of H*_S_* at the 50 m-isobath referred as H*_S,50_* (Table 1 in Supplementary Material). Finally, data of significant wave height were also extracted exactly on the ROP of each IPW. The values are therefore referred as H*_S,ROP_*

## Ethics Statements

This article did not make use of human or animal subjects, and its publication is approved by all authors.

## CRediT Author Statement

**Francesca Budillon:** Conceptualization, Data curation, Methodology, Software, Investigation, Writing – review & editing, Supervision, Funding acquisition; **Ines Alberico:** Conceptualization, Data curation, Methodology, Software, Investigation, Validation, Visualization, Writing – reviewing; **Sabrina Amodio:** Conceptualization, Data curation, Methodology, Visualization, Writing – original draft preparation, Reviewing & editing; **Pasquale Contestabile:** Data curation, Methodology, Software, Investigation, Validation, Visualization, Writing – Reviewing; **Sara Innangi:** Data curation, Visualization.

## Declaration of Competing Interest

The authors declare that they have no known competing financial interests or personal relationships that could have appeared to influence the work reported in this paper.

## Supplementary materials

Image, application 1
